# Biogenic Amines in Poultry Meat and Poultry Products: Formation, Appearance, and Methods of Reduction

**DOI:** 10.3390/ani12121577

**Published:** 2022-06-18

**Authors:** Wojciech Wójcik, Monika Łukasiewicz-Mierzejewska, Krzysztof Damaziak, Damian Bień

**Affiliations:** Institute of Animal Science, Warsaw University of Life Sciences, 02-786 Warsaw, Poland; krzysztof_damaziak@sggw.edu.pl (K.D.); damian_bien@sggw.edu.pl (D.B.)

**Keywords:** biogenic amines, poultry meat, chicken meat, food, quality

## Abstract

**Simple Summary:**

Meat consumption is on the rise, including poultry meat. With the storage of meat and the progressing process of food spoilage, the content of biogenic amines increases. Methods to prevent the formation of amines include: starter cultures, packaging methods, high hydrostatic pressure (HHP), ozonisation, radiation, use of essential oils, phytobiotics, and organic acids in food. The aim of this study was to compare the content of biogenic amines in poultry meat on the basis of the latest scientific reports and to present methods for preventing the formation of biogenic amines.The use of herbal extracts can not only reduce the occurrence of biogenic amines, but also improve production results and meat quality.

**Abstract:**

Poultry meat is a source of many important nutrients, micro- and macro-elements, and biologically active substances. During meat storage, many physicochemical changes take place, also affecting the content of biologically active substances, including biogenic amines.They are formed as a result of three processes: decarboxylation of amino acids by microorganisms, reductive amination, and transamination of aldehydes and ketones, and as a result of activity of body tissues. Excessive consumption of biogenic amines shows toxic properties. The increasing consumption of poultry meat and the lack of established limits for biogenic amine content is a major challenge for scientists, producers, and consumer organisations, which have not yet established limits for biogenic amine content in meat (including poultry meat). Analyses of biogenic amine content in meat account for less than 10% of scientific papers, which raises the scope of the problem of limiting biogenic amines in meat. Among the methods of amine reduction are methods of destroying or reducing microorganisms’ high hydrostatic pressure (HHP), ozonisation, radiation, or the use of essential oils.

## 1. Introduction

Meat is a key element of a balanced diet. Meat consumption has played a significant role in evolution, affecting the development of the human brain. What is more, it forms a source of wholesome and easily digestible protein, fatty acids, with a high contribution of polyunsaturated fatty acids of the omega-3 family, micro- and macro-nutrients (iron, selenium, potassium, magnesium, sodium, phosphorus, calcium), and group B vitamins. It is also a source of bioactive substances (carnitine, taurine, carnosine, and creatine) [1,2,3]. Increased production and consumption of meat is correlated with the improved financial situation of the population, which affects the demand for this type of food [4]. However, the mostpronounced increase of meat consumption primarily concerns developing countries [5].

Over the last decades, increased meat production has been observed globally, and the most intensive increase concerns poultry meat [6,7,8]. Poultry is highly popular among consumers. This stems from the absence of cultural contraindications for the consumption of poultry meat, its low production costs, and the high content of easily digestible protein [9]. Since 2000, the global meat production has increased by 47% (with poultry contributing to half of the increase), meaning the global production increased by 109 M tonnes. In 2018, the world’s meat production was 342 million tonnes, including 119.7 M tonnes of poultry [10]. The contribution of poultry meat in the global structure has grown from 69 M tonnes in 2000 to 119.7 M tonnes, and the increase of poultry meat production is envisaged, which in 2030 will reach the level of close to 151 million tonnes [10,11,12].Over the past decades, meat consumption has gradually increased. In 2000, the global meat consumption was 29.5 kg per person, while in the European Union, meat consumption was at 63 kg per capita. In 2019, global meat consumption was 34 kg per capita, while in the European Union it was 70.1 kg per capita. In 2000, world consumption of poultry meat was 9.8 kg per person, and Europeans consumed 22.1 kg per capita. In 2019, the global consumption of poultry meat was 14.7 kg per person, while Europeans consumed as much as 31.3 kg per person. According to forecasts, poultry meat consumption will continue to increase and will reach 15.1 kg per consumer globally and 33 kg per capita in European Union countries in 2029 [4,13].

## 2. Aim

The aim of the present study was to develop, based on the available literature and statistical data, an overview of the literature on the content of biogenic amines in poultry meat, their formation, changes occurring during meat storage, as well as the methods for limiting amine presence in poultry meat.

## 3. Poultry Meat Spoilage and Biogenic Amine Content

The chemical composition of meat depends on the animal species, age, genotype, nutrition, pre-slaughter treatment, and post-slaughter storage [1,8,14]. What is more, meat is one of the food types that undergo rapid spoilage. This process occurs at a faster rate for white meat than for red meat. This stems from the higher number of short fibres present in white poultry meat, which affects increased proteolysis. It is estimated that these changes occur between 4 and 10 days after slaughter [15,16,17]. The high protein content in poultry meat results in increased proteolysis and autolysis, which affects amino acid release (AA). Presence of AA and bacteria exhibiting decarboxylation capacity accelerates the process of meat spoilage and results in increased content of microorganism metabolites, including biogenic amines [9,18,19,20].

Meat spoilage or ageing also occurs in refrigeration conditions by microflora contamination during slaughter. It has been determined that poultry has a high slaughter contamination level, which further affects the processes that deteriorate the product shelf-life [9,21,22]. Early symptoms of meat spoilage are difficult to observe. Sensory assessment is insufficient due to its subjective nature. However, determination of microbial contamination is time-intensive and can be determined with the use of changes that occur in meat due to bacterial activity [7,21,23]. The changes determining meat ageing include: microbiological techniques, microscopic techniques, ionic changes, application of fluorescent spectroscopy, ion-mobility spectrometry (IMS), determination of changes in the basic composition of meat with near-infrared analysers (NIR),chemical changes (pH, total volatile basic nitrogen (TVBN), ATP, glucose, BAcontent), and modern techniques of electronic tongue or nose [9,14,23,24].

### 3.1. Biogenic Amines: Characteristics

Biogenic amines (BA) are compounds with a low molecular weight below 200 Da. They are formed through three processes: decarboxylation of amino acids by decarboxylation-capable microorganisms, reductive amination and transamination of aldehydes and ketones, or as a result of the activity of tissues in the organism. Furthermore, they can be accumulated in tissues throughout the life of the organism [25,26,27]. Biogenic amines can be divided (Table 1) in terms of the structure of the amino acid (precursor) into: aliphatic (putrescine, cadaverine, agmatine, spermine, spermidine), aromatic (tyramine, β-phenylethylamine), and heterocyclic (serotonine, histamine, and tryptamine), and for the number of amine groups: monoamines (tyramine, histamine, tryptamine), diamines (putrescine, cadaverine), and polyamines (agmatine, spermine, spermidine) [26,27,28]. What is more, endogenous and exogenous amines can be distinguished. Endogenous amines are mainly catecholamines, indoleamines, histamines, and BA of endogenous origin (spermine, spermidine, and low levels of putrescine and histamine), whereas exogenous amines are those formed mainly by the activity of microorganisms (cadaverine, putrescine, tyramine, histamine, β-phenylethylamine) [5,29].

Strains exhibiting decarboxylation activity include: *Enterobacteriaceae* (*Eschericha*, *Salmonella*), *Bacillus*, *Pseudomonas*, *Aeromonas*, *Clostridiaceae*, and mainly G-negative bacteria, some *Lactobacillus*, and Gram-positive, such as certain *Staphylococci* and *Enterococci* [18,21,22,30,31]. Buňkováet al. [18] analysed 88 strains of bacteria isolated from poultry skin and their decarboxylase activity. It was shown that numerous strains of *Enterobacteriaceae* and *Aeromonas* are characterized by decarboxylase-positive activity. Furthermore, certain *Lactobacillus* strains also demonstrate amino acid decarboxylation activity. In the case of poultry meat, it is believed that *Enterobacteriaceae* strains are the most common ones responsible for the increase of BA [8]. Strains differ in terms of the decarboxylation activity of specific amino acids, and a specific strain may contribute to the formation of a specific amine without the possibility of decarboxylation of other amino acids [32].

Biogenic amines are mainly found in protein-rich products (meat, fish, cheese) and in fermenting products. Amines most commonly found in poultry are: histamine (HIS), tyramine (TYR), cadaverine (CAD), and putrescine (PUT). Also present are β-phenylethylamine (PHM), spermine (SPM), and spermidine (SPD) [22,23,33,34].

### 3.2. Role of Biogenic Amines

Biogenic amines fulfil a range of important functions in live organisms, including human organisms [33]. They have been identified in both animal and plant tissues, as well as in eukaryotic organisms (bacteria, fungi) [28].

Biogenic amines are precursors for hormones, alkaloids, proteins, and nucleic acids, and are a source of nitrogen for the organism. Polyamines such as spermine, spermidine, and putrescine contribute to the natural growth of cells. These polyamines are also present in mammal sperm, fulfilling the role of gene expression modulators (binding with the locus in the DNA, activating genes or cell growth), supporting cellular growth of differential as well as initial embryologic development [26,28,35,36]. Excess of the aforementioned polyamines intensifies neoplastic degeneration, and their high contents were found in tumours [16]. Despite their positive action, an excess of BA exhibits toxic properties, and biogenic amines are known as toxic biomolecules [37]. Histamine is a widespread amine found in such organs as muscles, brain, intestines, stomach, uterus, or ovaries. Its action is related to H receptors (H1–H4) and it acts as a neurotransmitter and local hormone, modulating the activity of the stomach, work of the heart, smooth-muscle contraction, circadian rhythm, and maintaining body heat [26,28]. However, it exhibits a toxic effect when consumed in excess. Symptoms of the toxic effect of histamine include dilation and increased permeability of blood vessels, which results in ecchymosis, hives, itching, tingling, burning, headache, blood pressure drop, tachycardic responses, and breathing difficulties (airway constriction and hypoxemia). It further results in smooth-muscle contraction, resulting in diarrhoea, vomiting, and stomach-ache [16,38,39]. Histamine is strictly related to fish and fish product poisoning, but it also occurs in poultry meat, particularly during poultry meat processing in high temperatures and meat with skin processing [38,40]

On the other hand, tyramine is responsible for the reaction related to the consumption of excessive amounts of cheese, and its symptoms are referred to as “cheese reaction.” Its action resembles neurotransmitters, and it is characterized by the capacity for increased sympathetic activity of the cardiovascular system by releasing catecholamine (noradrenaline), which results in peripheral constriction of blood vessels and acceleration of the heart action (tachycardia), a blood pressure drop, and a blood glucose concentration increase. The influence on the formation of microhaemorrhages during blood vessel dilation results in inflammatory state formation [26,28,39]. In an organism, its presence can be detected in brain, spinal cord, heart, spleen, lungs, or kidneys [28].

Histamine and tyramine are referred to as psychoactive and vasoactive amines. Excessive intake of both histamine and tyramine results in acute allergic-like reactions, particularly from the nervous and cardiovascular system [26,39,41]. They are heterocyclic amines, which are linked to the neoplastic degeneration process. They are easily absorbable in the gastrointestinal tract, and an arilnitrenium ion is formed as a result of the reaction with cytochrome P450monoaminooxidase, which intensifies neoplastic-increasing processes during DNA replication. As presented by Plevaet et al. [40] in experiments of animals, they contributed to the formation of benign neoplastic lesions of liver and malignant ones in the large intestine.

Cadaverine and putrescine have a considerable impact on cell proliferation, including neoplastic cells. They accelerate neoplastic degeneration, producing changes within the oral cavity and tumour growth. Putrescine is an electrostatic amine, fulfilling many physiological roles, but it also exhibits the capacity to react with nitrites, forming heterocyclic nitroso-pyrrolidine with carcinogenic activity. They further intensify the toxic effect of the excess of heterocyclic amines (histamine and tyramine), which also exhibit the properties for reaction with nitrates, forming carcinogenic nitrosamines [15,26,30,42].

β-phenylethylamine acts as a neurotransmitter, which induces the release of dopamine, serotonin, and noradrenaline. It affects perception, memory, and behaviour. It was mainly found in the brain and spinal cord, but its excess, as withtyramine, affects the incidence of migraines and pressure drops [26,28,42]. When blood levels of β-phenylethylamine and tyrosine increase, an increase of other BA and type 2 diabetes may occur [43].

### 3.3. Biogenic Amines Index (BAI)

Amine content in meat is considered a meat freshness determinant. To this end, the quality index (QI) was developed—the sum of histamine, putrescine, and cadaverine divided by the sum of spermine and spermidine plus 1; subsequently, the biogenic amines index (BAI) was developed, which is the sum of histamine, tyramine, cadaverine, and putrescine [35]. The content of these amines increases with meat storage and their excessive consumption exhibits toxic effects, and thus the determination of amine content is important not only from the standpoint of meat freshness, but also for maintaining the health status of society (meat consumers) [5,27]. BAI is of high significance for the determination of amine content in cheese and meat because it includes the content of tyramine. BAI in fresh meat should not exceed 5 mg/kg, whereas the acceptable range with initial symptoms of spoilage is between 5 and 20 mg/kg. Meat with low hygienic quality is classified in the range 20–50 mg/kg, and spoiled meat has a BAI above 50 mg/kg [27].

### 3.4. Systemic Defensive Mechanisms

Natural defensive mechanisms of the organism, protecting against negative effects of consuming excessive levels of BA, are monoamine oxidase (MAO), diamine oxidase (DAO), and polyamine oxidase (PAO). However, this system is often disturbed by gastric problems of consumers, antidepressant intake, alcohol consumption, disruption of natural defensive mechanisms of the organism, or consumption of spoiled food containing high levels of BA. Additionally, the synergistic effect of these factors influences increased toxicity of biogenic amines. The main problem consists of MAO and DAO inhibitors, which include antidepressant drugs. It is estimated that 20% of the European population uses antidepressants, and the intake of this type of agent exhibits a growth tendency [27,44].

## 4. Monitoring and Recommended BA Consumption Standards

To care for the consumer health and to reduce the negative impact of consuming excessive amounts of BA, whose toxicity mechanism has not been fully understood, it is recommended to restrict consumption of BA-rich products [26]. International permissible limits of biogenic amine consumption are absent [28]. This issue has thus far been covered by numerous consumer organizations and food safety agencies. These include the European Food Safety Authority (EFSA), the Food and Drug Administration (FDA), the Food Safety Commission of Japan (FSCJ), and the World Health Organization (WHO). As a result of cooperation with the aforementioned entities and based on the Regulation EC/178/2002, the Rapid Alert System for Food and Feed (RASFF) database was created. However, the above organizations and the mentioned system mainly focus on the toxicity of histamine from fish and fish products [26,27,38,42]. Based on the FDA report, as provided by Rabieet et al. [30], the maximum level of tolerated histamine content in meat was determined as 100 mg/kg, whereas daily histamine consumption should not exceed 50 mg and 600 mg for tyramine. Danchuket et al. [42] reported that the permissible histamine level in healthy food should not exceed 50 mg/kg, the level between 50 and 200 mg/kg may have a harmful effect on consumer health, and the level above 200 mg/kg exhibits toxic properties. In turn, Feddern et al. [16] report that histamine and tyramine content above 100 mg/kg and β-phenylethylamine above 30 mg/kg show toxic effects on consumer health status. According to the EFSA Report, the daily recommended histamine intake is below 50 mg for healthy people, but below detection limits for people with a histamine intolerance, and600 mg of tyramine for healthy people who do not take drugs from the group of monoamine oxidase inhibitors (MAOI), but 50 mg for people taking third-generation MAOI drugs or 6 mg for people taking classic MAOI drugs. However, information on putrescine and cadaverine have proven insufficient in this scope [45].

## 5. Changes in the Content of Biogenic Amines in Poultry Meat

Numerous scientific reports have shown increased levels of tyramine, histamine, cadaverine, putrescine, and β-phenylethylamine in poultry with a concomitant decrease of spermine and spermidine levels [23]. Table 2 presents ranges of biogenic amine content in poultry meat based on the latest scientific reports and changes of biogenic amine content during storage. Table 2 shows changes in BA content with the duration of poultry meat storage (leg and breast muscles) in aerobic conditions, corresponding to the conditions of storage by consumers, and in modified atmosphere packaging (MAP). Based on the results presented by Min et al. [24] and Wojnowskiet et al. [34], it can be stated that the content of putrescine and cadaverine increased in a linear manner during the storage period, whereas in the case of BA detection at7 days (histamine, tyramine, and β-phenylethylamine), the values were lower than on days5 and 9 of storage.Furthermore, the tyramine content decreased in the pectoral muscles of ducks and quails. A linear increase in putrescine and cadaverine content wasobserved, with a decrease in spermine and spermidine content [8].

Based on Table 2, it can be observed the BAI (used to determine meat freshness and quality) increases with the level of BA. Discrepancies in the ranges provided by the authors may stem from the differences in the initial microbiological purity of meat and the differences resulting from the conditions and precision of methods used to determine BA [21].

### 5.1. Biogenic Amines Content in Poultry Products

The contents of BA in ready-to-eat products or products after heat treatment are presented in Table 3. Furthermore, cooked and shredded chicken breast muscles and smoked turkey were stored, and BA contents were determined with storage duration. Analyses by Hassan et al. [32] have shown that the levels of tyramine, putrescine, and cadaverine were at a level acceptable for consumption by the consumer. Histamine levels were not determined. The sum of determined BA for wings, thigh, and nuggets was 3.2, 5, and 7.8 mg/kg, respectively, for minimum analytical values, and 37.2, 42.2, and 68 mg/kg, respectively, for maximum values.

In the case of shredded cooked breast storage in aerobic conditions, higher values for the analysedBA were obtained than for storage of this meat in MAP conditions (30% CO_2_+70%N_2_). Higher oxygen accessibility affected the higher activity of decarboxylase, obtaining higher values for putrescine, cadaverine, and tyramine after 28 days of storage [14].

Ntzimaniet et al. [17] assessed changes of BA in smoked turkey breast fillet stored in aerobic conditions, vacuum, MAP (30% CO_2_ +70%N_2_), and in skin. Samples were stored for 30 days. The lowest value for histamine was determined in the fillets stored in skin (11.9 mg/kg), whereas the highest was for turkey fillets without skin stored in aerobic conditions (32.9 mg/kg). Low tyramine, putrescine, and cadaverine content was determined in both groups. Results obtained from MAP and vacuum groups did not indicate relatively high histamine and tyramine levels.

In a study by Iacuminet et al. [47], an increase in histamine and cadaverine was found during the maturation process of goose meat sausages. In one batch (spoiled sausages), high contents of histamine (415.3 mg/kg) and cadaverine (339.3 mg/kg) were found, whereas in the batch of sausages which were determined as unspoiled, the values of these amines were 5.6 and 32.1 mg/kgfor histamine and cadaverine, respectively.

### 5.2. Biogenic Amines vs. Poultry Health Status

Biogenic amines can be accumulated during life, and this accumulation mainly stems from the activity of decarboxylase-positive microorganisms occurring in the gastrointestinal tract [27]. Numerous publications have analysed the correlations between intestinal microbiota and the effect on production conditions. In the work of Tiihonenet et al. [49], Ross 308 chicken were provided with an addition of essential oils (EO) from thymol and cinnamaldehyde, which had a positive effect on production results and also on the stabilization of bacterial flora in the intestines. With the increase of EO content, the content of tyramine, putrescine, and tryptamine lowered in the cecum on day 41 of chicken fattening, whereas the levels of spermine and spermidine increased. Furthermore, the addition of EO affected the increased level of butyrate in the intestine, reduced propionic acid and isovaleric acid content on day 41 of rearing, and an increasedshare of *Lactobacillus* and *Escherichia coli* was found in the caecum. All disturbances of the digestive tract microbiota affect the maldigestion and amino acid absorption, which constitutes the substrate for the reaction of free amino acid decarboxylation and increased metabolites of protein fermentation in the caecum and the content of BA. Moreover, in the study cited by Apajalahti and Vienola [50] (Apajalahti and Badford (2000)), infection with *Eimeria maxima* also produced increased content of BA in the caecum, and only after 14 days was the level restored to the state from before the infection.On the other hand, addition of 0.2% inulin to broiler chicken feed resulted in a reduced caecum share of histamine by 26.6%, tyrosine by 27.8%, cadaverine by 18.4%, and putrescine by 11.6% [51]. Phytobiotic substances, such as herbal extracts from cinnamon, garlic, lemon, and rosemary present a positive effect on the poultry health status, protecting it from *C. perfringens* infection and from necrotizing enterocolitis in broiler chicks [46]. Biogenic amines further exhibit a toxic effect on the health status of chickens. Barnes et al. [52] analysed the influence of 0.1% and 0.2% histamine and 0.1% cadaverine addition and their mix of 0.1% each in a chicken diet. Metabolic pathologies were demonstrated, including gizzard erosion and proventricular ulcers (plaques), and they resulted in a lower final weight of chickens and an increased feed conversion ratio.

For the purpose of a better gastrointestinal tract development, putrescine injections in ovo were performed on day 17 of chick incubation at a dose of 0.05%, 0.1%, 0.15%, and 0.2%. Dosages of 0.05% and 0.1% influenced the increased weight of the gastrointestinal tract after hatching, however the intestine weight was reduced after 24 h post-hatching. Dosages above 0.1% exhibited toxic properties and reduced the number of hatched chicks [36].

## 6. Methods for the Detection of Biogenic Amines

Among the detection methods for BA, the following methods can be distinguished:

- High-performance liquid chromatography (HPLC),
- Gas chromatography (GC),
- Capillary electrophoresis (CE),
- Thin-layer chromatography (TLC),
- Fluorometric methods and enzymatic methods: enzyme-linked immunosorbent assay system (ELISA).

Based on the analysis of Ahmad et al. [26],it was shown that HPLC is the most popular method for the analysis of BA, accounting for 62% of the determinations carried out over a period of 5 years.

Then, electrochemical techniques (TLC and GC) accounted for 14%, fluorescence (ELISA) for 10%, capillary electrophoresis (CE) for 6%, and colorimetric and other methods for the determination of BA constitute 8%.

The greatest number of analyses for the determination of BA were performed in wine (16%), fish (13%), and urine (10%), while meat constituted only 8% as a product in which BAwere determined [26,27,28,29,30].

The determination of BA in Europe is usually performed by the HPLC method. This requires the complexity of two steps: the extraction of BA from the sample and the analytical determination of BA. Detectors (electrochemical, fluorescence, and ultraviolet (UV) detectors) are most commonly used. In the USA, spectrophotometric determination is the most common method for determining BA (mainly histamine). It is recommended by the Association of Official Agricultural Chemists (AOAC). It involves homogenisation in methanol, followed by filtration, use of anion exchange chromatography for separation, derivatisation, and the final step of spectrophotometric BA determination. The derivatisation reaction is an important step in that the determination of BA can be carried out with high sensitivity and strong retention. It accounts for 62% of the BA determination reaction.Problems during BA determination can arise from potential reactions of the food composition and interferences with other substances, and therefore purification of the sample is also an important step. The processes used are solid-phase and liquid-phase purification and its advanced version: dispersive liquid–liquid microextraction (DLLME), which accounts for about 13% of the reactions used for BA determination [26,28,29].

Among the extraction solvents used, acids (hydrochloric acid, trichloroacetic acid, and perchloric acid) and organic solvents (acetone, acetonitrile, methanol, perchloric acids, dichloromethane acid) are mainly used [28,29]. According to Ahmad et al. [26], among the most commonly used detectors for BA determination by HPLC was mass spectrometry (MS/MS), which accounts for as much as 34%. This was followed by fluorescence detector accounting for 29%, and UV detector, for 16%.

In the work of Wojnowskiet et al. [24], dispersive liquid–liquid microextraction combined with gas chromatography-mass spectrometry (DLLME-GC-MS) and an electronic nose model were used for BA determination. The prototype electronic nose had a dedicated sample chamber, which was used for rapid analysis of the volatile fraction of chicken meat samples, based on headspace analysis and fingerprinting. An artificial neural network based on machine learning was developed, resulting in good accuracy of measurement and calculation of the coefficient of determination. The results of BA determination by DLLME-GC-MS and the electronic nose method were then compared. It was shown that an artificial neural network(electronic nose) with an appropriately determined regression coefficient can be used to determine BA in meat—in this case poultry meat.

## 7. Methods for Restricting Biogenic Amines Content in Poultry Meat

Due to the toxic effect of BA, the scientific circles face an important challenge consisting in restricting their occurrence. Methods for limiting the level of BA include:

- Starter cultures,
- Packing methods,
- High hydrostatic pressure (HHP),
- Ozonation,
- Radiation,
- Use of essential oils, phytobiotics, and organic acids in foods [5,28,41,53].

To reduce the level of BA and eliminate microorganisms with decarboxylation capacity, starter cultures and probiotic strains are used, which do not show such capacity. They are mainly used in fermenting and long-maturing products. Both single strains as well as the synergistic effect of several strains are used. Those strains are mainly lactic acid bacteria: *Lactobacillus sakei*, *Lactobacillus curvatus*, and *Lactobacillus plantarum* [37,41]. Moreover, strains exhibiting the capacity for biogenic amine oxidation to aldehydes or ammonia are also used. These include *Micrococcus varians*, *Natrinemagari*, *Brevibacterium linen*, *Lactobacillus sakei,* and *Lactobacillus curvatus* [5].However, sometimes, some strains within the same family also show decarboxylation capacity, which does not affect the reduction of BA. It is important to select a pure strain for the starter culture. It also happens that the starter culture strain is not able to control the “wild” strain present on the meat. The “wild” strain is the strain showing the ability to decarboxylate amino acids [5,21].

Meat is packed using several methods, under aerobic conditions, vacuum, active packaging (AP), and modified atmosphere packaging (MAP). Alternative methods of meat packing relative to meat storage in aerobic conditions are used to prolong the product shelf-life and limit the formation of BA. MAP is used with a mixture of different gases (CO_2_, N_2_, CO, Ar). Fraqueza et al. [22] demonstrated a reduction of the presence of BA in turkey meat stored in the conditions of different MAP gas mixtures compared toin aerobic conditions. Similar results were obtained by Rodriguez et al. [14], whereas higher levels of BA (histamine and tyramine) under vacuum and MAP conditions were obtained by Ntzimani et al. [17].

Use of high hydrostatic pressure (HHP) results in microorganism inactivation, and a change of microorganism structure, genome, and morphology, without impacting the product quality, thus prolonging the shelf-life of the product.The effect of HHP depends largely on the pressure level, treatment time, time of application, and type of food. The analysis of restricting the BA level in sausage based on poultry meat (Alheira) subject to high hydrostatic pressure has shown that the pressure of 600 MPa for 960 s results in limited levels of BA in poultry products without impacting the sensory properties of the product [54].

Ozonation also has a positive impact on microorganism inactivation in foods [55]. Mercogliano [56] examined the effect of ozone (O_3_) on the content of BA. The influence of ozone decontamination on the content of BA was demonstrated. Ayranciet et al. [55] demonstrated a considerable reduction of microorganisms and physicochemical changes of turkey breast meat (pH, colour). What is more, water-holding capacity and cooking yield increased.

The use of radiation is a method used for surface product decontamination. It is used to decontaminate numerous food products, including meat and meat products. However, an excessive radiation dose may affect the physicochemical properties of meat, and it changes the structure and properties of decarboxylase enzymes [5,57]. Lázaro et al. [58] analysed different UV doses (0.62, 1.13, 1.95 mW/cm^2^) and stored at 4 °C for 9 days. For the correlation between exposure time on restricting the content of BA in chicken meat, 1.95 mW/cm^2^ for 90s turned out to be most efficient in preserving food and limiting BA, andfurthermore stabilized pH and L*,a*,b* parameters. Similar results were obtained byMin et al. [19] using a dose of 2 kGy and comparing results of biogenic amine content to organic acids (0.2 M acetic, citric, and lactic acid). Organic acids do not affect the structure and physicochemical properties of meat. A positive influence was shown on the elimination of *Campylobacter spp*., the main cause ofhuman campylobacteriosis, peracetic acid (PAA) on carcasses packed in MAP, and storage for 12 days. Bertram et al. [53] demonstrated a considerable reduction of putrescine and cadaverine in turkey breast muscles.

Application of phytobiotics and phytobiotic substances with other biogenic amine reduction methods may produce a synergistic effect. Poultry meat in the active packaging (AP) system with the addition of *Rosmarinus officinalis* essential oil at 4% reduced the level of BA as compared with a control group of poly-coupled packaging (PP). After seven days of storage, the analysis revealed lower values for putrescine, β-phenylethylamine, cadaverine, and histamine, and a higher tyramine level [25].The literature includes numerous studies on the effect of phytobiotics on meat quality improvement and the reduction of biogenic amine content. Such substances include curcumin, thymol, piperine, capsicum, garlic, green onion, red pepper, cloves, ginger, cinnamon, cassia, and fennel extracts [28,58].The following spices have been used in dried sausage production to reduce the biogenic amine content: star anise, amomumtsao-ko, clove, cassia, fennel, bay leaf, and nutmeg. Cassia and fennel extracts exhibited the most pronounced effect on reducing BA [58].

Grape pomace in the broiler chicken diet had a positive effect on meat quality, lipid profile, and oxidative stability, and reduced the content of BA in experimental groups relative to the control group after 7 days of storage: putrescine by 25.9%, 37%, and 44.4% for groups with 2.5%, 5%, and 7% addition of grape pomace, respectively. The tyramine level was reduced by 27.7% for groups with a 2.5% and 7% addition and increased by 11.1% for the 5% addition group. Thecadaverine level was below detection limits in experimental groups [33].

## 8. Conclusions and Future Perspectives

The issue of food safety has been influencingconsumers for many years, who currently prefer low-processed food, with a small number of additives, safe, and above all, healthy, including the acceptable level of BA. The toxicity of excessive consumption of BA is a serious challenge for food safety, which is a global problem for producers, processors of meat products, food technologists, scientists, and consumers. Meat (in general) represents only 8% as an object of analysis for determination of BA, which is a very small proportion of the total structure and shows an increase in the control of meat, including poultry meat [14,21,59].

The use of modern methods (e.g., electronic nose)for the detection of BA may in the future constitute a popularization of the determination of these compounds in many public products, including poultry meat and meat products. Modern methods, unlike traditional analytical methods, are less time-consuming, they do not require the use of many expensive chemical reagents, no highly qualified personnel areneeded to carry out analyses, and they can be helpful to determine the content of BA in real time [42]. The search for new, fast, and universal methods of the amine level determination can contribute to popularizing the determination of these compounds and to increasing consumer awareness on the toxicity of amines. The ongoing changes in the monitoring of food and toxicity of BA are important from the point of view of consumer health [26].Today, food monitoring should be geared towards producing safe products (with lower levels of contamination), while providing reliable information to all consumers (including those with dietary restrictions, especially those at risk of damaging MAO and DAO mechanisms) so that they can make informed choices.

## Figures and Tables

**Table 1:** Classification of biogenic amines [28].

Biogenic Amine	Structural Formula	Classification	Precursor
Putrescine	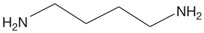	Diamine Aliphatic	Ornithine
Cadaverine	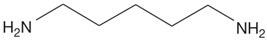	Diamine Aliphatic	Lysine
Agmatine	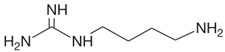	Polyamine Aliphatic	Arginine
Spermine	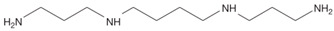	Polyamine Aliphatic	Arginine Ornithine
Spermidine	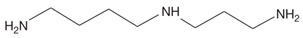	Polyamine Aliphatic	Arginine Ornithine
Tryptamine	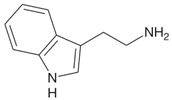	Monoamine Heterocyclic	Tryptophane
β-phenylethylamine	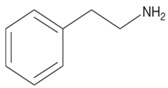	Monoamine Aromatic	Phenylalanine
Tyramine	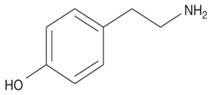	Monoamine Aromatic	Tyrosine
Histamine	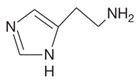	Monoamine Heterocyclic	Histidine

**Table 2:** Contents of biogenic amines (HIS: histamine, TYR: tyramine, PUT: putrescine, CAD: cadaverine, BAI: biogenic amine index, SPM: spermine, SPD: spermidine, and PHM: β-phenylethylamine) in chicken meat during storage under different storage conditions (mg/kg).

Sample	Storage Conditions	Day	HIS	TYR	PUT	CAD	BAI	SPM	SPD	PHM	Source
**Chicken breast**	**Air**	1	ND–1.48	ND–1.3	0.8–58.3	ND–19.8	0.8–80.88	38.8–53.3	7.9	ND	[7,19,24,46]
		3	4.2–6.2	3.2–5.5	1.1–2.7	1–24.8	9.5–39.2	60.2	6.3	ND	
		5	2.3–7.7	4.1–5.7	1.8–75.5	10.5–10.5	18.7–99.5	41.4–63.7	5.7–7.3	1.6	
		7	16.7	3.8	49.5	3.8	73.8	53.2	7.6	4.3	
		9	9.4	130.5	207	91.1	438	77.4	7.5	4.7	
		14	8.6	2.9	300.3	160.6	472.4	37.1	5	NA	
		17	19.2	4	409.6	252.7	685.5	36.6	4.8		
	**MAP**	1	ND	ND–0.3	ND–48	ND–8.5	ND–56.8	17.7–56.6	7.5–13.2	NA	[7,15]
		3	ND	ND	ND	ND	ND	17.3	7.3–7.6		
		5	ND	1.4	58.4	25.4	85.2	39.2	11.6		
		8	ND	2.3	65.3	30.8	98.4	38.3	10.7		
		9	1.8	ND–1.9	26.4–72.5	8.5–21.7	36.7–104.2	16.0–39.2	5.9–10.7		
		14	1.6–14.5	ND–6.0	29.8–248.9	9.5–120.6	40.9–390	37.3	8.7		
		17	26.8	8.9	354	223.7	613.4	31.5	7.8		
**Chicken legs**	**Air**	1	ND	3.7	0.3	1.6	2.03	46.6	6.8	0.4	[19]
		3	4.4	4.4	1.3	3.4	13.6	92.4	15.3	0.1	
		5	5.4	6.4	7.8	3.6	23.1	70.5	9.1	10.3	
		7	11.9	7.2	13.7	6	38.9	84.7	8.2	5.9	
		9	8.3	46.7	163.6	40.3	259.1	68.9	9.6	2.5	
**Quail breast**	**Air**	1	NA	356.8	ND	ND	NA	9.4	7.1	NA	[8]
		3		272.3	2.4	ND		5.1	6.8		
		5		177.9	5.8	3.8		2.2	6.7		
		7		106.7	9.2	7.0		1.8	6.6		
		9		93.5	13.9	10.1		1.8	6.6		
		11		50.6	11.9	19.6		1.5	6.5		
		13		28.2	15.9	17.7		1.5	6.3		
		15		20.6	17.0	16.6		1.5	6.3		
		17		1.7	17.7	16.3		ND	6.3		
**Duck breast**	**Air**	1	NA	135.5	0.2–3.4	ND	NA	10.4–49.4	7.7–10.5	NA	[8,46]
		3		92.1	0.4	ND		9.8	6.4		
		5		49.9	31.0	4.4		7.5	6.8		
		7		27.0	34.7	4.3		1.8	7.2		
		9		13.0	54.8	4.2		1.5	6.3		
		11		8.5	18.2	3.8		1.5	6.3		
		13		4.4	13.2	3.3		1.5	6.4		
		15		3.9	12.7	1.3		1.5	6.3		
		17		2.4	8.2	ND		ND	6.6		
**Duck thigh**	**Air**	1	NA	NA	3.0	NA	NA	24.1	5.8	NA	[47]
**Duck liver**	**Air**	1			5.2			70.0	30.9		

ND—not detected; NA—not analysed/not available.

**Table 3:** Content of biogenic amines (HIS: histamine, TYR: tyramine, PUT: putrescine, CAD: cadaverine) in different poultry products(mg/kg).

Sample	Days of Storage	HIS	TYR	PUT	CAD	Source
**Wings**	1	1.3–20.1	1–10.3	-	0.9–6.8	[32]
**Thigh**	1	2.6–20.5	1.4–12.2	-	1–9.5	
**Nuggets**	1	3.9–28.4	1.8–21.9	-	2.1–17.7	
**Shredded cooked breast** **aerobiosis packaging**	1	-	3.2	0.2	-	[14]
	28	-	4.9	58.7	23.1	
**Shredded cooked breast** **MAP: 30% CO_2_ + 70%N_2_**	1	-	3.2	0.2	-	
	28	-	3.9	23.2	3.2	
**Smoked turkey breast fillet** **stored in air**	1	ND	ND	ND	ND	[17]
	14	8.7	1.8	1.3	0.6	
	30	32.9	2.5	2.5	1.8	
**Smoked turkey breast fillet under vacuum**	1	1.7	0.5	0.8	1.4	
	14	5	1	1.9	1.1	
	30	15.6	12.5	2	2.5	
**Smoked turkey breast fillet in skin**	1	4.9	ND	ND	0.2	
	14	6.8	0.6	0.4	1.2	
	30	11.9	4.3	1	2.5	
**Smoked turkey breast fillet** **MAP 30% CO_2_+70%N_2_**	1	ND	ND	ND	ND	
	14	1.9	0.5	0.5	2.4	
	30	14.9	10.2	2.1	4.5	
**Unspoiled goose sausages**	ND	5.6	ND	ND	32.1	[48]
**Spoiled goose sausages**	ND	415.3	ND	ND	339.3	

ND—not detected.

## Data Availability

The data presented in this study are available on request from the corresponding author.
